# Aortic aneurysm disease versus aortic occlusive disease: differences in postoperative ICU requirements after open elective abdominal aortic surgery

**DOI:** 10.1186/cc11077

**Published:** 2012-03-20

**Authors:** J Bisgaard, HK Jørgensen, T Gilsaa, E Ronholm, P Toft

**Affiliations:** 1 Littlebaelt Hospital Kolding, Denmark; 2 Odense University Hospital, Odense, Denmark

## Introduction

Open elective abdominal aortic surgery is a high-risk procedure involving clamping of the aorta. Indications include abdominal aortic aneurysm (AAA) or aortic occlusive disease (AOD) causing lower limb ischaemia. These patients are often regarded as one entity in postoperative study settings. However, previous studies indicate that risk profiles, inflammatory activity, and haemodynamic capacity may differ between these groups [1,2]. The aim of this study was to evaluate postoperative ICU requirements after open elective abdominal aortic surgery, hypothesising that AAA patients had longer ICU stays and needed more mechanical ventilation or acute dialysis than did patients with AOD.

## Methods

This cohort study was based on prospectively registered data from the Danish National Vascular Registry and the Danish ICU Database between 1 January 2007 and 1 May 2010. The study population comprised all patients (*n *= 1293) undergoing open elective, primary aorto-iliac bypass, or aorto-femoral bypass procedures (*n *= 363) or abdominal aortic aneurysm repair (*n *= 930) in the eight hospitals performing these procedures in Denmark. The primary endpoints were: ICU stay >24 hours, mechanical ventilation, and acute dialysis.

## Results

Patients in the AAA group were older (70 ± 7 vs. 62 ± 9 years, *P *< 0.001), predominantly males (80 vs. 49%, *P *< 0.001), with a higher prevalence of preoperative cardiac co-morbidity (34 vs. 24%, *P *= 0.001), and malignant disease (2.7 vs. 0.6%, *P *= 0.02). In contrast, AOD patients had a higher prevalence of smoking (95 vs. 86%, *P *< 0.001), and diabetes (16 vs. 9%, *P *< 0.001). AAA patients had larger intraoperative blood losses (1,610 (1,000 to 2,500) vs. 1,200 (750 to 1,800) ml, *P *< 0.001), but duration of surgery was shorter (161 (130 to 205) vs. 194 (160 to 240) minutes, *P *< 0.001). Postoperatively, more AAA patients had ICU stays >24 hours (62 vs. 45%, *P *< 0.001), tended to need mechanical ventilation more often (16 vs. 12%, *P *= 0.08), and more needed acute dialysis (3.8 vs. 0.9%, *P *< 0.03).

## Conclusion

Compared to the AOD group, more AAA patients had ICU stays >24 hours and more often needed acute dialysis. Distinguishing between these two diseases may be useful in planning and distribution of ICU resources. Furthermore, considering these two patient groups as different pathological entities may be advised in future studies.
